# Naturally-occurring tooth wear, tooth fracture, and cranial injuries in large carnivores from Zambia

**DOI:** 10.7717/peerj.11313

**Published:** 2021-04-20

**Authors:** Blaire Van Valkenburgh, Paula A. White

**Affiliations:** 1 Department of Ecology and Evolutionary Biology, University of California, Los Angeles, Los Angeles, California, United States; 2 Center for Tropical Research, Institute of the Environment and Sustainability, University of California, Los Angeles, Los Angeles, California, United States

**Keywords:** Carnivore, Craniodental, Kafue, Lion, Leopard, Luangwa Valley, Spotted hyena, Zambia

## Abstract

Determining the incidence and causes of craniodental damage in wild carnivores is often constrained by limited access to specimens with associated ecological data, such as prey type and abundance. We assessed dental condition and cranial injuries in lion, leopard, and spotted hyena in relation to prey and predator populations in Zambia’s Luangwa Valley, where large prey are more abundant and lion and leopard more numerous, and the Greater Kafue Ecosystem, where smaller prey species are more prevalent and lion and leopard less common. In Luangwa, lions had significantly higher rates of tooth fracture, and blunt trauma injuries attributable to prey-handling, compared to Kafue lions. In contrast, leopards in both regions had similar rates of tooth wear and breakage. Overall, lions showed a significantly higher tooth fracture rate than leopards on a per tooth basis. Spotted hyenas had the highest rates of tooth wear and fracture among all three carnivores, and greatly exceeded previously recorded rates based on historical samples. Despite larger numbers of lion and leopard in Luangwa, there was no difference in incidence of intraspecific injuries between regions. These results are consistent with a greater abundance of large prey species, especially buffalo, in the diets of Luangwa lions, and previous work showing a reliance on smaller prey species in Kafue throughout the large carnivore guild.

## Introduction

Large (>21 kg) carnivores lead a risky life. Because of their size, they need to kill prey that are as large, or larger, than themselves (Carbone et al., 1999; Carbone, Teacher & Rowcliffe, 2007). Their typical prey, ungulates, are difficult to subdue, wielding weapons (hooves, horns, antlers) that can and do injure their attackers, fracturing their limbs and skulls in some cases (e.g., Rausch, 1967; Creel & Creel, 2002; Mukherjee & Heithaus, 2013). The teeth of large carnivores are often broken, due to stresses incurred while killing prey, consuming bone, and in combat with conspecifics as well as other competitors (Van Valkenburgh, 1988, 2009; Mann, Van Valkenburgh & Hayward, 2017). Undoubtedly, the risks of being a large carnivore have shaped their evolution, but we have little data on the frequency and severity of injuries sustained by large predators. Injury incidence is difficult to quantify from field observations; broken bones mend and cracked teeth are not easily seen from a distance. Nevertheless, skeletal and dental trauma is often recorded in the bones and teeth of individuals, and surveys of natural history collections have provided insights into the frequency and distribution of trauma in extinct and extant carnivores (e.g., Wobeser, 1992; Wilkins et al., 2007; Binder, Thompson & Van Valkenburgh, 2009; Losey et al., 2014; Brown et al., 2017; Collados, Garcia & Rice, 2018; Tong et al., 2020).

When considering craniodental injuries, free-ranging specimens are required because dental pathologies, tooth wear, and fracture vary significantly in captive versus wild carnivores (Wenker et al., 1999; Curtis et al., 2018). Museum collections represent valuable archives of skeletal material for comparative osteological and dental investigations, but historic specimens often lack logistical and/or natural history information (De la Sancha, Boyle & Patterson, 2017), limiting interpretation of results. As an alternative source of contemporary specimens, many species of carnivores are legally hunted, and the associated permitting procedures require reporting of relevant data such as date and location. However, hunted specimens are usually sequestered in private collections, making them difficult to access and consequently they remain underutilized for purposes of scientific investigations.

Here, we report on tooth wear, tooth breakage, and cranial injuries in free-ranging lion (*Panthera leo*), leopard (*P. pardus*), and spotted hyena (*Crocuta crocuta*) from two regions, the Luangwa Valley (LV) and the Greater Kafue Ecosystem (GKE) in Zambia between 2007 and 2012. Cranial material was obtained primarily from hunted specimens with the exception of two lions that were killed by Zambia’s Department of National Parks and Wildlife (DNPW) as problem animals owing to threats to humans or livestock. Tooth wear and fracture were recorded for each specimen, as well as evidence of antemortem osteological pathologies such as blunt trauma and bite marks, indicative of injuries potentially associated with prey-handling and intraspecific conflict, respectively.

Previous work on dental trauma in large carnivores suggests that, within age classes, variation in tooth wear and tooth breakage among species is largely reflective of diet, especially bone consumption. Species such as spotted hyenas that frequently consume large bones have higher rates of tooth fracture and heavier wear than species that consume less bone, such as cheetahs (*Acinonyx jubatus*) (Van Valkenburgh, 1988, 2009; Mann, Van Valkenburgh & Hayward, 2017). Moreover, tooth fracture frequency within a species is likely to increase as large carcasses are consumed more completely, a feeding behavior that is likely to be favored when prey are difficult to acquire (Mech & Frenzel, 1971; Carbone et al., 2005; Vucetich, Vucetich & Peterson, 2012). A recent comparative study of extant gray wolf (*Canis lupus*) populations found that wolves from regions or time periods characterized by high numbers of prey had significantly less tooth wear and fracture, while wolf populations with reduced prey availability were associated with greater carcass utilization, higher bone consumption, and more tooth damage (Van Valkenburgh et al., 2019).

Moreover, predators confront greater danger from injury when tackling large prey species (Rausch, 1967). In Zambia, the LV and GKE differ significantly in the relative abundance of prey, in particular, very large (>500 kg) herbivore species with the former region having greater numbers of Cape buffalo (*Syncerus caffer*), hippopotamus (*Hippopotamus amphibius*), and elephant (*Loxodonta africana*) as well as a small population of endemic Thornicroft’s giraffe (*Giraffa camelopardalis thornicrofti*) (DNPW, 2016; IUCN, 2016), and each of these species factor importantly into the diet of LV lions (Zambian Carnivore Programme unpublished, in Creel et al. (2018)). In contrast, small to medium-sized herbivores are currently the most numerous prey species in GKE (DNPW, 2016; Creel et al., 2018). Therefore, we hypothesized that capturing, killing, and feeding (including scavenging) on very large prey might result in elevated tooth fracture rates, as well as increased incidents of blunt trauma in LV than GKE carnivores of comparable ages. Lions (IUCN, 2006) and possibly leopards (Rosenblatt et al., 2016) are also more numerous in LV than GKE, and increased intraspecific competition and conflict in LV might be reflected in a greater number of cranial injuries indicative of fighting, such as bite wounds and claw marks.

Our purpose in this paper is to explore the array and frequency of craniodental injuries that are apparent in three species of large African carnivores in each of two regions in Zambia.

## Materials & methods

### Study area

**Figure 1:**
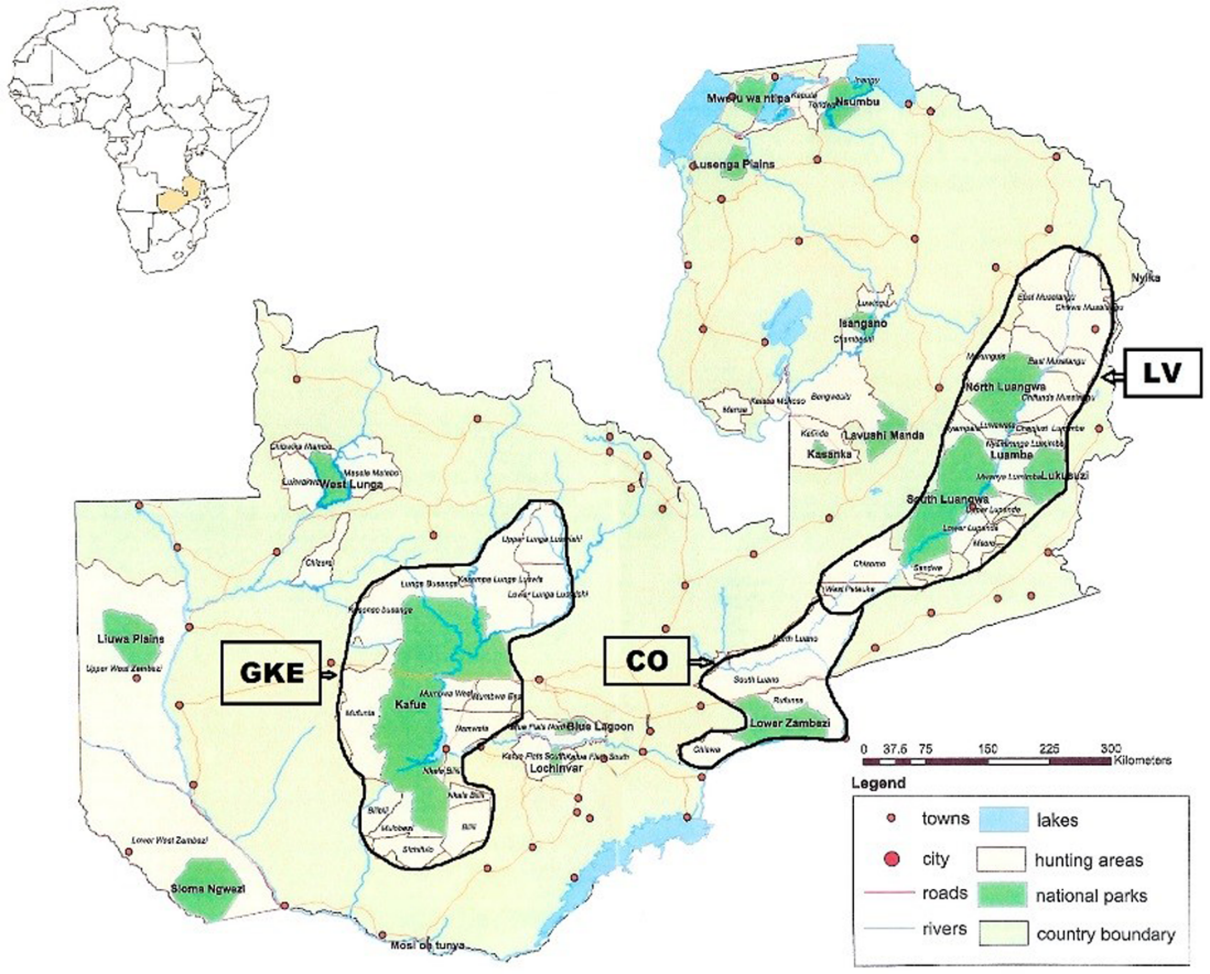
Map showing location of Luangwa Valley and Greater Kafue Ecosystem in Zambia. Location map of Zambia in southcentral Africa showing the Luangwa Valley (LV) and the Greater Kafue Ecosystem (GKE). The corridor area (CO) consists of contiguous habitat between the Luangwa Valley and Lower Zambezi river.

The Republic of Zambia occupies 752,614 km^2^ in southcentral Africa within 15° 00′ S and 30° 00′ E. Samples were obtained from two regions, Luangwa Valley (LV) in the east and the Greater Kafue Ecosystem (GKE) in the west (Fig. 1). Both regions are comprised of national parks (NPs) and adjacent game management areas (GMAs). The LV contains four NPs totaling 15,630 km^2^ that, combined with the adjacent GMAs, covers 62,038 km^2^ (Astle, 1999). The GKE is an approximately 66,547 km^2^ area comprised of Kafue NP (KNP) (22,400 km^2^) and adjacent GMAs (Siamudaala, Nyirenda & Saiwana, 2009). There are no fences or other barriers, and animals routinely cross between NPs and GMAs. Legal trophy hunting for lion, leopard, and spotted hyena occurs in most GMAs. Two lions killed by the wildlife authority (DNPW) as problem animals that were 10–15 km outside of LV (*n* = 1) and GKE (*n* = 1) were assigned to the geographically closest region.

### Obtaining specimens

Between 2007 and 2012, one of us (PAW) examined and photographed skulls and teeth of hunted lion, leopard, and spotted hyena as part of a larger study on age estimation of trophy hunted lions (White & Belant, 2016; White et al., 2016). Skulls of hunted carnivores were made available by professional hunters and taxidermists. Skulls of two lions killed as problem animals were made available by DNPW. All osteological material was examined and photographic data collected in Zambia in partnership with DNPW (formerly Zambia Wildlife Authority, Research/Employment Permit No. #008872). A total of 118 lion (LV 60; GKE 58), 45 leopard (LV 18; GKE 27) and 13 spotted hyena (LV 6; GKE 7) skulls were evaluated in this study. Due to post-mortem damage or incomplete photographic views, not all lion skulls could be used in every analysis. Thus, samples sizes for lion varied slightly as follows: tooth wear and breakage 115 lions (LV 58; GKE 57); cranial injury 116 lions (LV 60; GKE 56).

Lion hunting in Zambia is restricted to males. Sex of sampled lions was confirmed from hides or trophy photographs. We restricted our sample of problem lions to include only males. Leopard hunting in Zambia is restricted to males, and sampled leopards were assumed to be males. However, trophy photographs or hides were not available for all sampled leopards, and the possibility cannot be excluded that some hunted individuals were females (Spong, Hellborg & Creel, 2000). Because spotted hyena are encountered less frequently, the species is taken more opportunistically and hunter selectivity is lower than for lion or leopard. Consequently, the spotted hyena sample likely consisted of both female and male specimens. For each of the three species, we assumed that hunter bias in selectivity was similar among the two regions.

### Photographs

We captured digital images of each skull using a Nikon 35mm D3300 digital camera with Nikkor AF 70–300 mm f/4.5-6.3 lens (Nikon USA Inc., Melville, NY, USA). We photographed skulls with the mandible articulated from left and right lateral sides, anterior, occipital and dorsal views. Thereafter, we disarticulated the mandible and inverted the cranium to obtain a palatal view and dorsal and lateral views of the mandibles. Digital images were organized by a field ID number assigned to each specimen and stored on SD cards for later examination on a computer and display monitor.

### Age estimation of samples

Because the probability of having broken a tooth or suffered an injury increases with age, it is important to control for age differences between samples from the two regions. We assigned age classes to each sampled lion, leopard, and spotted hyena based on published standards (below) that represent the best available methods of age estimation for each species. We assigned specimens to one of three age classes initially with skulls in-hand and verified age assignments later using digital images.

In lions, crown wear of the upper second premolar (P2) has been shown to increase with age (Schaller, 1972; Smuts, Anderson & Austin, 1978; White & Belant, 2016). We visually scored P2 crown wear as: (1) no wear/sharp, (2) moderate wear/rounded, or (3) heavy wear/flat, and used P2 scores in conjunction with overall tooth wear features (following Smuts, Anderson & Austin, 1978; Miller et al., 2016) in assigning each lion to one of three age classes: young adult < 5 years; mature adult ≥ 5–7 < years; and old adult ≥ 7+ years (Fig. S1). Assignment to the mature adult age class was confirmed in most cases by the partially to fully obliterated closure of the interfrontal suture after Smuts, Anderson & Austin (1978) (Fig. S2). We assigned each leopard to an age class based on wear of the upper and lower canines (C1, c1) and premolars (P3, p3,4) following the criteria of Stander (1997) with the modification that younger age classes were combined as follows: young adult ≤ 4 years; mature adult 5–6 years, and old adult 7–10 years (Fig. S3). We assigned spotted hyena to one of three age classes (young 1–3 years; mature > 3–6 years; old > 6–16 years) based on wear of the lower third premolar (p3) after Kruuk (1972) (Fig. S4). We tested for differences in age structure of sampled lion and leopard from LV vs. GKE using chi-square performed in SPSS v.26 with significance levels set at *P* < 0.050.

### Scoring of dental condition

For the regional comparisons, we scored tooth breakage and overall wear from digital photographs alone. Scoring of tooth breakage and wear was performed subsequent to, and independent of, age estimation, and involves a more comprehensive review of wear across the entire tooth row as opposed to focusing on one or a few specific teeth (e.g. canines, premolars). Notably, PAW did the age estimation and BVV did the subsequent dental wear and fracture analyses, blind to the age estimates. As in earlier studies (Van Valkenburgh, 1988; Van Valkenburgh & Hertel, 1993; Van Valkenburgh, 2009), breakage was scored by tooth position and type, i.e., incisors, canines, premolars, and molars. A tooth was counted as broken in life (antemortem) only if there was clear evidence of subsequent wear on the tooth following breakage (Fig. S5). Teeth broken at time of death or post-mortem with apparently unworn, sharp edges were not counted as broken and were excluded from analyses.

We assigned one of five tooth wear stages to each individual: (1) little or no apparent wear observed on shear facets or blunting of cusps, (2) shear facets apparent on carnassial teeth and cusps slightly blunted on some teeth, (3) shear facets apparent on carnassial teeth and cusps blunted on most teeth, (4) carnassial teeth exhibiting strong shear facets and moderate blunting of premolar cusps, or (5) carnassial teeth exhibiting strong shear facets and/or blunted cusps, and premolars and molars with well-rounded cusps. The five categories were then reduced to three for the analysis, (1) “slight” (stage 1 only), (2) “moderate” (stages 2 and 3), and (3) “heavy” (stages 4 and 5) (Fig. S6).

We summed tooth wear stage distribution and fracture frequencies by species and location (LV or GKE) and, for lion and leopard, compared distribution of wear stage between regions using chi-square with significance set at *P* < 0.050. Interspecific comparisons using the entire dataset were made on (1) number of broken teeth as a percentage of the total number of teeth, (2) percentage of individuals with one or more broken teeth, and (3) percentage of tooth fracture by tooth position (incisors, canines, premolars, carnassials). For lion and leopard, the percentage of broken teeth and breakage by tooth position for each species were compared between samples from LV and GKE using chi-square with significance set at *P* < 0.050. Sample size of spotted hyena (*n* = 13) was insufficient to compare between the two regions.

We then compared the results of tooth damage in modern Zambia carnivores to historical samples for all three species obtained from various museums as described in Mann, Van Valkenburgh & Hayward (2017) using chi-square with significance set at *P* < 0.050. Due to methodological differences in assessing tooth wear, i.e., Zambia samples from photographs; historical samples in-hand, we restricted our comparisons of modern with historic specimens to tooth breakage. Further, it should be noted that our Zambia felid sample consists of males, whereas Mann, Van Valkenburgh & Hayward (2017) present data for the sexes combined. In addition, the historical samples span more years (several decades) than our 6-year Zambia study, and thus are not strictly comparable.

### Scoring of cranial injuries

We examined skulls for evidence of past injury including healed or healing fractures or other pathologies excluding those directly associated with dental breakage, i.e., missing bone at the alveolar margin of a broken tooth. Damages that were associated with time of death were readily determined, e.g., freshly splintered bone, and excluded from analyses. We initially assessed cranial injuries with skulls in-hand and subsequently quantified damages using digital images.

**Figure 2:**
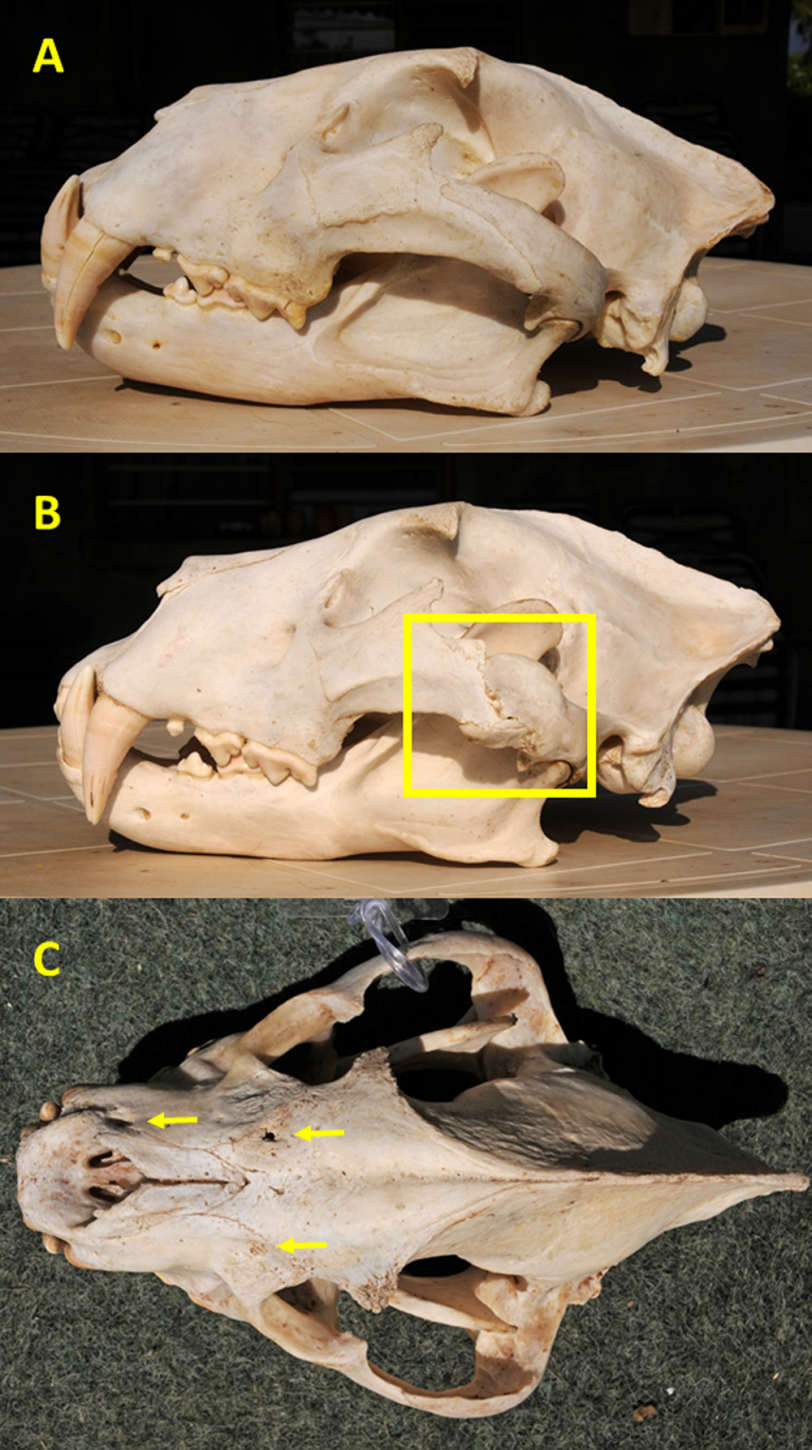
Examples of cranial injuries found in Zambia carnivores. (A) Lion skull with undamaged zygomatic arch. (B) Lion skull with major, partially-healed fracture and remodeled zygomatic arch (shown in square) likely caused by blunt trauma from a large prey. (C) Spotted hyena skull with one puncture and two circular depressions (arrows) from bites to the frontal, maxilla, and premaxilla bones. Photo credits: Paula A. White.

We scored injuries as resulting from either blunt trauma or intraspecific conflict as follows: (1) major fractures, defined as fractures that caused misalignment of one or more bones sometimes resulting in remodeling, and shallow depressions or gouges with irregular shapes were presumed to have resulted from blunt trauma sustained during prey capture and handling, such as a kick or impact of a horn boss; or (2) deep circular depressions and linear scratches, consistent with bite and claw marks, respectively, were judged to have been caused by intraspecific conflict (Fig. 2). Because some judgements were subjective, it is possible that prey-inflicted injuries vs. intraspecific conflict were erroneously assigned in some instances. Major fractures most commonly involved the zygomatic arches (Fig. 2) or nasals, while other injuries were found primarily on the premaxilla, maxilla, nasal, frontal, and parietal bones.

We recorded the number of incidents of traumas per skull, with an incident defined as occurrence of one type of pathology per animal because it could not be known if multiple injuries were inflicted during a single event. For example, a lion with one or more fractures (which could have occurred during a single predation event) was counted as one incident of blunt trauma attributable to prey-handling, and a lion with one or more bites and scratches (which could have occurred during a single lion fight) was counted as one incident of intraspecific trauma, whereas a lion with one or more fractures, one bite mark and deep scratches was counted as two incidents (fracture(s) = one blunt trauma; bite and scratches = one intraspecific trauma).

### Carnivore populations

From the literature, we obtained lion population size estimates during the sampled time period of 400–750 in LV (0.0064–0.012 lions/km^2^) with an additional approximately 50 lion inhabiting the CO, and an estimated 250–500 lion in GKE (0.0037–0.0075 lions/km^2^) (IUCN, 2006). We used IUCN (2006) estimates rather than available focal studies in reporting lion densities because the former represent the larger regions inclusive of the NPs and all adjoining GMAs. Lion appeared to be both more numerous and present at higher density in LV than in GKE (IUCN, 2006).

The LV is considered as having one of the highest densities of leopard in Africa (Gregg, 2019) although few empirical data exist on Zambia’s leopard populations (Purchase, Mateke & Purchase, 2007). A study conducted from 2006–2008 in a 491 km^2^ area of the LV reported leopard densities of 3.36 to 4.79 leopards/100 km^2^ (Ray, 2011). Subsequent investigations (2012–2014) in a 313 km^2^ area of the LV estimated from 5.08 to 8.50 leopards/100 km^2^ (Rosenblatt et al., 2016). We found no current publications reporting on leopard density in GKE although there is no evidence to suggest that leopard density in GKE was as high, or higher, than in LV.

Scant data are available on the current status of spotted hyena populations in LV or GKE (Purchase, Mateke & Purchase, 2007); available reports suggest that the species occurs at relatively low density in both regions. Based on camera traps, spotted hyena appeared to be approximately 2–5 times less abundant than leopard in Ray’s (2011) LV study area. Playback censuses conducted in 2003–2004 by Carlson, Carlson & Bercovitch (2004) estimated 18–44 spotted hyenas/1,000 km^2^ in northern Kafue National Park (NKNP) and commented that spotted hyena were shy and rarely seen. Likewise, Creel et al. (2018) reported on spotted hyena in NKNP between 2013–2016 as present but not common. This is consistent with reports of spotted hyena occurrence in the larger GKE between 2007–2012 where sightings were relatively infrequent and most often consisted of singles or pairs (White & Kim, 2018). These limited and localized results highlight the need for updated surveys on the status of spotted hyena in LV and GKE (Purchase, Mateke & Purchase, 2007).

### Prey populations

From the literature, we obtained relative abundance of prey in each region. Aerial surveys conducted by DNPW in 2015 contributed to population trend data for 12 species of large mammals over the time periods 2002–2015 in LV and 2006–2015 in GKE (DNPW, 2016). Population estimates for 12 key prey species (DNPW, 2016), as well as elephant (IUCN, 2016), and hippopotamus (Chansa & Milanzi, 2010) are provided as Supplemental Information. Because all three carnivore species consume carrion, we augmented estimates of elephant population size with counts of elephant carcasses, where available (CITES Secretariat, 2016). Additionally, we referenced Creel et al. (2018) for relative abundance of prey species in NKNP from 2013–2016.

## Results

### Age structure of sampled carnivores

When considering samples from the Luangwa Valley (LV) and Greater Kafue Ecosystem (GKE) combined, the majority of lion (81%) and leopard (73%) were mature adults, i.e., ≥5 years old, with 11% of lions and 13% leopards having estimated ages of ≥7 years, i.e., old adults (Table 1) (Data S1). There was no significant difference in the age distribution between regions either for lion (*X*^*2*^ = 1.701, df = 2, *P* = 0.427) or leopard (*X*^*2*^ = 2.639, df = 2, *P* = 0.267). The 13 spotted hyena specimens included young (31%), mature (23%), and old (46%) individuals (Table 1) (Data S1).

**Table 1:** Age structure of sampled Zambia carnivores.

Sample	*N* (*%*)	Young adult	Mature adult	Old adult
*Panthera leo* (Luangwa)	60	3 (*5*)	49 (*81.7*)	8 (*13.3*)
*Panthera leo* (Kafue)	58	6 (*10.3*)	47 (*81*)	5 (*8.6*)
*Panthera pardus* (Luangwa)	18	3 (*16.7*)	11 (*61.1*)	4 (*22.2*)
*Panthera pardus* (Kafue)	27	3 (*11.1*)	22 (*81.5*)	2 (*7.4*)
*Crocuta crocuta* (all Zambia)	13	4 (*30.8*)	3 (*23.1*)	6 (*46.1*)

### Tooth wear and fracture frequency between species

**Figure 3:**
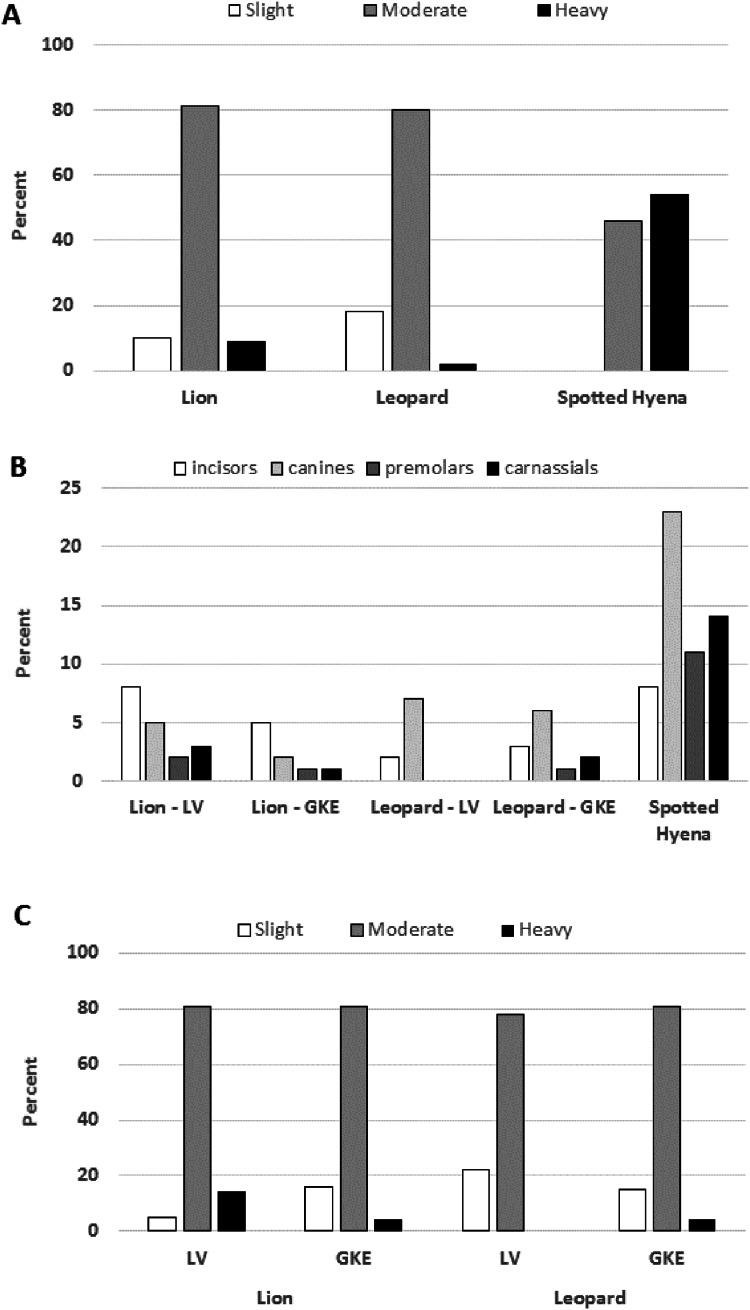
Tooth wear stage and tooth fracture by position in Lion, Leopard and Spotted Hyena. (A) Percent of individuals assigned to each tooth wear stage in the three species sampled in Zambia. Total sample size is 115 lions, 45 leopards and 13 spotted hyena. (B) Percent of teeth broken for each tooth position within each sample by region, Luangwa Valley (LV), Greater Kafue Ecosystem (GKE). Sample sizes for spotted hyenas were too small to compare regions. For tooth sample size, see Table S1. (C) Percent of individuals assigned to each tooth wear stage within each region. Total sample size is 58 (Lion-LV), 57 (Lion-GKE), 18 (Leopard-LV), and 27 (Leopard-GKE).

The distribution of individuals among tooth wear categories was similar between lions and leopards, with lions showing a tendency towards having more individuals in the heavy wear class (Fig. 3A) (Data S2) despite the two species having similar age distributions (Table 1). However, lions showed twice the tooth fracture rate of leopards on a per tooth basis (4% vs. 2%; *X*^*2*^ = 8.150, df = 2, *P* = 0.002) (Table 2) (Table S1) (Data S2). Although lions also exceeded leopards in the percentage of individuals with one or more broken teeth (53% vs. 44%, Table 2), this difference was not significant (*X*^*2*^ = 0.957, df = 2, *P* = 0.211). Across the tooth row, lion had significantly more broken incisors and premolars than leopard (*P* < 0.030) but the two species did not differ greatly in their rates of canine or carnassial tooth fracture (Fig. 3B) (Table S1) (Data S2).

**Table 2:** Tooth fracture in Zambia carnivores from two regions and comparisons with historical samples.

Sample	*N* (skulls)	*N* (teeth)	% individuals w/≥1 brkn tooth	% broken teeth	% broken teeth w/canine teeth excluded
*Panthera leo* (Mann, Van Valkenburgh & Hayward, 2017)	133	3,491	33.8	3.5	2.8
*Panthera leo* (all Zambia)	115	3,286	53	3.8	4
*Panthera pardus* (Mann, Van Valkenburgh & Hayward, 2017)	146	3,697	39	3.7	2.4
*Panthera pardus* (all Zambia)	45	1,313	44.4	2.1	1.5
*Crocuta crocuta* (Mann, Van Valkenburgh & Hayward, 2017)	179	5,084	51	4.3	3.5
*Crocuta crocuta* (all Zambia)	13	402	76.9	11.7	10
**Within Zambia**					
*Panthera leo* (Luangwa)	58	1,658	62.1	5.0	5.0
*Panthera leo* (Kafue)	57	1,628	43.9	2.6	2.6
*Panthera pardus* (Luangwa)	18	517	38.9	1.9	1.0
*Panthera pardus* (Kafue)	27	796	48.1	2.3	1.5

As expected based on their fondness for consuming bone, spotted hyena had more tooth wear and higher tooth fracture rates than lion or leopard. Among the hyenas sampled, there were none that exhibited only slight tooth wear, and the majority were placed in the heavy wear category (Fig. 3A) (Data S2). On a per tooth basis, spotted hyena had a fracture rate of 12%, which is three times that observed for lion (4%, *X*^*2*^ = 50.117, df = 2, *P* < 0.001) and six times that observed for leopard (2%, *X*^*2*^ = 71.223, df = 2, *P* < 0.001) (Table 2) (Table S1). On a per individual basis, 77% of the spotted hyena had at least one broken tooth, whereas that proportion was 53% for lion and 44% for leopard (Table 2) (Data S2). The higher tooth fracture observed in spotted hyena was distributed across the tooth row, with canine teeth and cheek teeth being more likely to be broken than incisors (Fig. 3B).

### Tooth fracture frequency within species

Sample sizes of lion and leopard were sufficient to explore differences in tooth fracture frequency between populations in LV and GKE. Among the lions, the differences between the two regions were striking with LV lion exhibiting significantly higher fracture frequencies than their GKE counterparts, both in percentage of broken teeth (5% vs. 2.6%, *X*^*2*^ = 13.213, df = 2, *P* < 0.001) and percentage of individuals with one of more broken teeth (62.1% vs. 43.9%; *X*^*2*^ = 3.827, df = 2, *P* = 0.038) (Table 2) (Data S2). Notably, the higher fracture frequency of LV lions was apparent across the entire tooth row, from incisors to carnassials (Fig. 3B) (Table S1). In association with their higher tooth fracture rates, LV lions also had more rapid rates of tooth wear than GKE lions, as evidenced by a smaller proportion of individuals in the slight wear class along with a larger proportion of individuals in the heavy wear class (Fig. 3C; *X*^*2*^ = 6.623, df = 2, *P* = 0.036), despite the fact that the age distributions of lions did not differ between LV and GKE (Table 1). Unlike the lions, the leopards from the two regions did not differ significantly in fracture frequency on either a per tooth (*X*^*2*^ = 0.161, df = 2, *P* = 0.424) or per individual basis (*X*^*2*^ = 0.375, df = 2, *P* = 0.381) (Fig. 3C) (Table S1) (Data S2).

### Comparison with historical samples

Tooth fracture rates on a per tooth basis in male lion and leopard from Zambia were largely comparable to those observed in historical samples of both sexes of these species that originated from different parts of Africa as published in Mann, Van Valkenburgh & Hayward (2017) (Table 2). There were significant differences between the historical and Zambia samples in a few cases. Among lion, the proportion of individuals with at least one broken tooth was significantly greater in the Zambia lion (53% vs. 33.8%; *X*^*2*^ = 4.322, df = 2, *P* = 0.025) as was the proportion of teeth fractured in LV lion (5% vs. 3.5%; *X*^*2*^ = 6.717, df = 2, *P* = 0.007) but not GKE lion. The per tooth fracture rate for Zambia leopard was significantly less than that recorded by Mann, Van Valkenburgh & Hayward (2017) (2.1% vs. 3.7%; *X*^*2*^ = 8.641, df = 2, *P* = 0.003). The most striking difference is in the spotted hyena. Our small sample (13) collected over 6 years in Zambia broke their teeth much more frequently on both a per tooth basis (12% vs. 4%; *X*^*2*^ = 44.816, df = 2, *P* = 0) and per individual basis (77% vs. 51%; *X*^*2*^ = 694.932, df = 2, *P* = 0) than the spotted hyena sample in Mann, Van Valkenburgh & Hayward (2017) that included 179 skulls spanning decades and multiple countries.

### Cranial injuries

Over half (68/116; 59%) of all our sampled lions had at least one cranial injury ascribed to natural causes, and 16 individual lions (14%) showed evidence of both blunt trauma and intraspecific conflict (Data S3). LV lion had significantly higher rates of blunt trauma injuries attributable to prey-handling compared to lion in GKE (52% vs. 30%) (*X*^*2*^ = 5.422, df = 2, *P* = 0.019) (Table 3). Among the 31 LV lions with blunt trauma injuries, 15 consisted of major healed or healing fractures (Fig. 2) as opposed to only five lions with major injuries in GKE. In contrast, the incidence of injuries ascribed to intraspecific conflict was similar for lion from LV and GKE (32% vs. 30%) (Table 3).

**Table 3:** Frequency of individuals with at least one injury ascribed to either blunt trauma or intraspecific conflict in lion and leopard by region and in spotted hyena (both regions).

Sample	*N* (%)	Blunt trauma *major fractures*^1^		Intraspecific *bite wounds, scratches*	Non-specific	Total incidents
*Panthera leo* (Luangwa)	60	31 (51.7) *15 (25)*		19 (31.7)	0	50 (83.3)
*Panthera leo* (Kafue)	56	17 (30.4) *5 (8.9)*		17 (30.4)	0	34 (60.7)
*Panthera pardus* (Luangwa)	18	4 (22.2)		8 (44.4)	1 (5.6)	13 (72.2)
*Panthera pardus* (Kafue)	27	5 (18.5)		11 (40.7)	5 (18.5)	21 (77.8)
*Crocuta crocuta*	13	N/A		N/A	4 (30.8)	4 (30.8)

**Note:**

1 Subset of the 31 (LV) and 17 (GKE) blunt traumas in lions that consisted of major fractures.

Among leopard, the number of incidents attributable to blunt trauma and intraspecific conflict combined were similar between regions (LV 72%; GKE 78%) (Table 3) (Data S3). No major fractures were seen in leopards. Due to small sample sizes and the number of incidents that could not be assigned to specific categories, differences in the frequency of blunt trauma injuries related to prey-handling vs. intraspecific conflict in LV vs. GKE leopards were not conclusive (Table 3).

Of the 13 spotted hyena skulls examined, four exhibited signs of significant natural trauma, including one with multiple penetrating bite wounds across its rostrum which may have been inflicted by a lion or a hyena (Fig. 2). Because the likely causes of the natural injuries in spotted hyena (prey-handling vs. intraspecific conflict vs. lion) could not be determined to a reasonable degree, all were classified as non-specific (Table 3) (Data S3).

### Prey diversity, abundance and trends

Prey species diversity was similar in both regions with a few exceptions; a small population of endemic Thornicroft’s giraffe occurs only in LV, and lechwe *Kobus leche* occur only in GKE. Relative abundance and density of prey varied between regions and among species (DNPW, 2016) (Data S4). Buffalo (DNPW, 2016), elephant (IUCN, 2016), and hippopotamus (Chansa & Milanzi, 2010) occurred in much higher abundance and density in LV compared with GKE. Data on elephant carcasses were available only from a specified survey area in LV where from 4 to 49 carcasses per year (mean of 19 carcasses/year) were reported from 2002–2012 (CITES Secretariat, 2016) (Data S5). Hippopotamus surveys conducted between 2005–2008 found that LV contained the largest concentration in Zambia, with the Luangwa river accounting for 62% (25,000) of the country’s overall hippopotamus population compared to 10% (4,000) in the Kafue river in GKE (Chansa & Milanzi, 2010).

Higher numbers of several species of small to medium-sized antelopes (e.g., lechwe, puku *K. vardonii*, waterbuck *K. ellipsiprymnus*, sable *Hippotragus niger*) were recorded in GKE (Data S4). Impala *Aepyceros melampus* were the most abundant antelope by far in both systems, although density of puku and sable were also high in GKE. Lechwe populations in GKE were highly localized in the northwest corner of the ecosystem (DNPW, 2016). A slightly different picture of prey composition in Kafue is given by Creel et al. (2018) who reported the species of highest densities in northern Kafue National Park (NKNP) as puku, impala, and warthog (*Phacochaerus aethiopicus*). Aerial surveys indicated that for the majority of key wildlife species in GKE, wildlife numbers that had generally declined from the mid to late-1990s increased over the period 2006–2012. Similarly, over the period 2002–2012 in the LV, numbers of most wildlife species were reported to be stable or increasing (DNPW, 2016) (Data S4).

## Discussion

Wild carnivores experience increased levels of natural tooth wear (Bodecker, 1925; Van Horn, McElhinny & Holekamp, 2003; Curtis et al., 2018) and tooth breakage with age (Van Valkenburgh, 1988, 2009). Despite the fact that the age distribution of lions was similar in Luangwa Valley (LV) and Greater Kafue Ecosystem (GKE), we found significantly higher rates of tooth wear and fracture in LV lions. Because the methods of assessing tooth wear differed (photographs vs. specimens in-hand), we were unable to compare tooth wear between modern and historic carnivores. However, the fact that rates of tooth breakage in historic samples of African lion were similar to modern lion in GKE affirms that actual differences in tooth fracture rates exist between modern carnivores inhabiting Zambia’s two regions.

A higher proportion of LV lions showed moderate/heavy tooth wear, and the percentage of lions with more than one broken tooth was 18% greater compared with lions from GKE. Tooth breakage in LV lions was higher across all tooth types, but this was more pronounced at the canines (5% in LV vs. 2% in GKE) and incisors (8% in LV vs. 5% in GKE). Previous observations of carnivore feeding behavior documented associations between tooth type employed and specific tissue type being consumed and found that incisors and canines (to a lesser degree) are used to pull muscle and skin from a carcass whereas posterior teeth, i.e., carnassials, are more often used when consuming skin, or muscle plus bone (Van Valkenburgh, 1996). Tooth breakage rates increase when carcass utilization is higher (Van Valkenburgh et al., 2019). Higher breakage rates among all tooth types in LV lions suggests that they may have been consuming prey more completely and consequently consuming more bone than lions in GKE.

Greater carcass utilization and thus increased tooth wear and breakage has been correlated with declines in the ratio of numbers of prey (elk (*Cervus canadensis*) or moose (*Alces alces*)) to predators (gray wolves) in both North American and Scandinavian ecosystems (Van Valkenburgh et al., 2019). Our study lacked sufficient empirical data to thoroughly examine predator/prey ratios that may have differentially influenced tooth wear and breakage. However, limited prey availability does not appear to explain the higher rates of tooth fracture in LV vs. GKE lions.

A more plausible explanation is that tooth wear and breakage varied as a consequence of the prey species being targeted. Cape buffalo, a preferred prey for lion (Hayward & Kerley, 2005), occurred at far greater density in LV than in GKE, as did elephant and hippopotamus (Chansa & Milanzi, 2010; DNPW, 2016). Moreover, Creel et al. (2018) reported that buffalo recently (2013–2016) made up only 10% of the diet of lions in northern Kafue National Park (NKNP) (significantly less than the 30% reported in GKE by Mitchell, Shenton & Uys (1965) five decades earlier) and that lion diet in NKNP now consisted primarily of small to medium-sized herbivores. This is in stark contrast to lion in LV where 60% of the diet is comprised of large-bodied species, i.e., buffalo, hippopotamus, elephant, and giraffe (Zambian Carnivore Programme unpublished, in Creel et al., 2018). Buffalo are particularly challenging prey with extremely tough skin >1 cm thick (Schaller, 1972) and are among the most dangerous quarry for lion to hunt and kill (Hayward & Kerley, 2005). Canine teeth are critical to the killing bite, and are prone to fracture if subjected to bending loads such as might occur when teeth contact bone (Van Valkenburgh & Ruff, 1987). The largest sharpest cheek teeth (carnassials) are likely critical for opening carcasses (Van Valkenburgh, 1996). Consumption of more tough-skinned, large-boned prey likely contributed to the higher overall rate of tooth wear and breakage found in LV lions.

Additional support for the concept that LV lions are tackling larger, more perilous prey comes from our cranial injury data. More than half (52%) of the LV lions sampled had suffered blunt trauma to their skull, including 15 lions with major skull fractures that likely resulted from impacts with the hooves or horns of their prey. The combination of elevated canine tooth and skull fracture rates in LV lions may be a result of killing and scavenging large prey. Alternatively or in addition, the higher rates of canine tooth breakage in LV vs. GKE lions may be due to higher lion density (IUCN, 2006) that could have resulted in increased intraspecific conflict (and breakage of canines during fights) in the former. However, lions in both regions exhibit similar rates of cranial bite and scratch mark injuries suggesting no significant difference in levels of lion-lion conflict among regions.

Among leopards, tooth wear and breakage rates showed slight variation by tooth type in LV and GKE, but no significant difference between regions. This finding suggests that prey species of comparable size are taken by leopard in both regions, and is consistent with recent reports of small to medium-sized herbivores representing primary prey of leopards both in NKNP (Creel et al., 2018) and LV (Ray, 2011).

In contrast to lions, the incidence of cranial injuries in leopards occurred at roughly the same frequency in LV and GKE. No major cranial fractures were seen among leopards. The lack of empirical data on leopard population sizes in either region, in addition to the number of injuries that could not be confidently assigned to a specific cause precluded further analyses of cranial injuries in leopards.

Consistent with prior work (Van Valkenburgh, 2009), interspecific differences in rates of tooth fracture corresponded to levels of bone consumption; spotted hyena exhibited the most dental damage, followed by lion and leopard. Notably, the Zambia hyenas exhibited the highest rate of tooth fracture recorded for this species, more than doubling previous estimates from museum specimens. We have yet to find an explanation for these high rates of wear and fracture. Spotted hyena are known to hunt and scavenge a wide variety of prey (Hayward, 2006), although scavenging of large numbers of elephant carcasses might have been an important factor during this study. In GKE, spotted hyenas returned to chew on the bones of an elephant carcass 35 days after its death (White & Diedrich, 2012). In Botswana, spotted hyena were found to scavenge on an elephant carcass for as long as 50 days (Cozzi et al., 2015). Availability of greater numbers of elephant carcasses due to natural mortality, deaths resulting from human-wildlife conflict, and poaching may have resulted in high rates of dental attrition due to the difficulty of slicing elephant hide and/or consumption of larger quantities of bone by hyena, and by lion in LV relative to GKE, although we need comparative data on elephant carcass availability from GKE to draw this conclusion.

Comparisons of our data on tooth fracture in extant populations of lion, leopard, and spotted hyena with previously published data for historical samples yielded mixed results, and should be viewed with some caveats. The historical samples include individuals from multiple locations in Africa and, within a species, tooth wear can vary with location due to substrate (e.g., lions; Smuts, Anderson & Austin, 1978). Moreover, Mann, Van Valkenburgh & Hayward’s (2017) historic samples include individuals collected over a span of decades, and samples that span longer time intervals are likely to produce lower rates of tooth fracture than those that span shorter intervals because tooth fracture rates can vary over time depending on resource availability and longer intervals are likely to include periods of minimal tooth fracture (Mann, Van Valkenburgh & Hayward, 2017). Consequently, we might have expected that our Zambia samples that span six years would produce higher estimates of tooth fracture frequency than those presented in Mann, Van Valkenburgh & Hayward (2017) collected between 1889 and 1967.

Nevertheless, in most cases, our tooth fracture rates did not differ greatly from those reported in Mann, Van Valkenburgh & Hayward (2017), with two exceptions. Lions from LV had much higher rates of tooth fracture per individual (62% vs. 34%) and somewhat higher per tooth (5% vs. 3.5%) than the historical sample means. The difference at the individual level is especially striking and suggests that LV lions are unusual in showing such a high prevalence of broken teeth. The difference might reflect the fact that we sampled only males whereas Mann, Van Valkenburgh & Hayward (2017) included both sexes, but this seems an unlikely explanation given that our sample of GKE males had rates of fracture (44%) similar to those of the historical samples. The second exception is the extremely high rates of tooth wear and fracture in our sample of 13 Zambia spotted hyena relative to historical samples (12% vs. 4% on a per tooth basis, and 77% vs. 51% on an individual basis). Examination of a larger sample of skulls from LV and GKE would be of interest to see if the results from this study are truly representative of hyenas from these two regions in Zambia, or if other explanations are needed for the extreme tooth wear and breakage recorded here.

In our introduction, we suggested that studies of cranial and dental injuries in large carnivores could enhance our understanding of the forces that mold their morphology and behavior, beyond what can be seen from field observations of living individuals. Over half of the lions we sampled had experienced one or more cranial injuries which would not have been easily detected in vivo. This substantial incidence of injury suggests that their skulls are certainly not overbuilt for expected loads and may represent a compromise between maximizing strength and limiting mass. Similarly, the prevalence of broken teeth indicates that the evolution of tooth structure, shape, and size is the result of multiple, sometimes opposing demands (e.g., sharpness vs. fracture resistance). Beyond providing us with insights into the long term selective forces that shaped these carnivores, rates of tooth wear and fracture frequency can give us information about current conditions that impact individual fitness. For example, tooth wear and fracture likely increase when food becomes limiting, and bone and carcass utilization rise. In Zambia, the very high levels of dental attrition and breakage in the spotted hyena suggest that these populations may somehow be food limited. Lions of LV suffer more dental fracture and more severe cranial injuries than those of the GKE, which we propose may be due to hunting larger, more dangerous prey, and/or greater carcass utilization. Resolving this would require more detailed data on prey choice and feeding behavior of carnivores in the two regions.

Our findings of injuries in LV lions raise the question: why risk hunting large prey when smaller prey are available? Buffalo occur at much higher abundance in LV than GKE, and the preference of lions for hunting buffalo (Mitchell, Shenton & Uys, 1965; Schaller, 1972) and other large game is well-documented (Hayward & Kerley, 2005). This preference is especially pronounced during the dry season when lack of cover likely contributes to a lowered success at stalking the faster, smaller ungulates (Mitchell, Shenton & Uys, 1965; Hayward & Kerley, 2005). Although hunting large prey carries heightened risks (Hayward & Kerley, 2005), the benefit is procurement of a substantial amount of meat and is consistent with optimal foraging theory based on energetic return for effort (Pyke, Pulliam & Charnov, 1977). In addition, cub growth is influenced greatly by nutrition (Smuts, Robinson & Whyte, 1980), and cubs are more likely to obtain meat from large vs. small kills (Schaller, 1972). Thus, from an evolutionary standpoint, the increased risk to a lion of sustaining a cranial injury while securing large-bodied prey is offset by enhanced cub survival as well as energetic return.

## Conclusions

Our survey of craniodental injuries of trophy hunted lion, leopard, and spotted hyena has revealed some intriguing differences among and within species, and underscores the value of utilizing trophy specimens in empirical research. Undoubtedly, our ability to interpret the differences would be enhanced by additional surveys of these species in other localities, especially those with metadata on predator and prey numbers and carnivore feeding preferences. Still, our low-cost, low-tech survey captured significant information from a sample of trophy hunted individuals, a study group that remains an underutilized resource for scientific investigations.

We recommend that collection and archiving of standardized photographs of carnivore teeth and skulls be incorporated into existing trophy monitoring programs to allow for more comprehensive investigations on naturally-occurring injuries and dietary stress in carnivore populations, including comparisons across different regions and time periods. Additional data on the health and condition of wild carnivores are always welcome, and relatively simple surveys of craniodental trauma are an easy add-on to the conservation biology tool box.

## Supplemental Information

10.7717/peerj.11313/supp-1 Supplemental Information 1 Illustration of tooth wear stages of the P2 in Lion used to assign individuals to age class.

10.7717/peerj.11313/supp-2 Supplemental Information 2 Examples of the degree of closure of the interfrontal suture of Lion used to assign individuals to age class. Photo credit: P.A. White.

10.7717/peerj.11313/supp-3 Supplemental Information 3 Illustration of tooth wear stages of the C1, c1, P3, p3,4 in Leopard used to assign individuals to age class.

10.7717/peerj.11313/supp-4 Supplemental Information 4 Illustration of tooth wear stages of the p3 in Spotted Hyena used to assign individuals to age class.

10.7717/peerj.11313/supp-5 Supplemental Information 5 Antemortem and post-mortem tooth breakage. Supplemental Figure S5. (A) Lion dentition showing (1) natural shearing wear on posterior edge of P4 and (2) upper canine tip that was broken while lion was alive and worn smooth from subsequent use. (B) Lion dentition showing sharp, unworn edges of breakage to the posterior edge of P4 (arrow) and upper canine (arrow) from damage that occurred after death. Photo credits: Paula A. White

10.7717/peerj.11313/supp-6 Supplemental Information 6 Examples of tooth wear stages. Supplemental Figure S6. Lion mandibles showing examples of the three tooth wear stages. (A) Heavy wear. (B) Moderate wear. (C) Slight wear. Photo credit: Paula A. White.

10.7717/peerj.11313/supp-7 Supplemental Information 7 Tooth counts and fracture rates by tooth position. Total number of teeth present for each tooth position and percent broken in parentheses for individuals from Luangwa Valley (LV) and the Greater Kafue Ecosystem (GKE).

10.7717/peerj.11313/supp-8 Supplemental Information 8 Age estimates and assigned age classes of sampled Lion, Leopard, and Spotted Hyena from Zambia.

10.7717/peerj.11313/supp-9 Supplemental Information 9 Tooth wear stage, tooth counts, and tooth fracture by position of sampled Lion, Leopard, and Spotted Hyena from Zambia.

10.7717/peerj.11313/supp-10 Supplemental Information 10 Incidents and types of cranial injuries of sampled Lion, Leopard, and Spotted Hyena from Zambia.

10.7717/peerj.11313/supp-11 Supplemental Information 11 Prey abundance, density, and trends in Luangwa Valley (LV) and Greater Kafue Ecosystem (GKE) obtained from aerial surveys (DNPW, 2016).

10.7717/peerj.11313/supp-12 Supplemental Information 12 Elephant carcass counts collected from 2002-2012 at a Monitoring of Illegal Elephants (MIKE) site in Luangwa Valley (CITES Secretariat, 2016).

## Additional Information and Declarations

### Field Study Permissions

The following information was supplied relating to field study approvals (i.e., approving body and any reference numbers): Paula A. White held a Research/Employment Permit No. #008872 while conducting research as the Zambia Lion Project in partnership with Zambia Wildlife Authority ZAWA (since renamed the Department of National Parks and Wildlife DNPW).

### Data Availability

The following information was supplied regarding data availability: The raw data are available as Supplemental Files.

## References

[ref-1] Astle WL (1999). A history of wildlife conservation and management in the mid-Luangwa Valley, Zambia.

[ref-2] Binder WJ, Thompson EN, Van Valkenburgh B (2009). Temporal variation in tooth fracture among Rancho La Brea dire wolves. Journal of Vertebrate Paleontology.

[ref-3] Bodecker CF (1925). A consideration of some of the changes in the teeth from young to old age. Dental Cosmos.

[ref-4] Brown C, Balisi M, Shaw CA, Van Valkenburgh B (2017). Skeletal trauma reflects hunting behaviour in extinct sabre-tooth cats and dire wolves. Nature Ecology & Evolution.

[ref-5] Carbone C, Frame L, Frame G, Malcolm J, Fanshawe J, Fitzgibbon C, Schaller G, Gordon IJ, Rowcliffe JM, Du Toit JT (2005). Feeding success of African wild dogs (*Lycaon pictus*) in the Serengeti: the effects of group size and kleptoparasitism. Journal of Zoology.

[ref-6] Carbone C, Mace GM, Roberts SC, Macdonald DW (1999). Energetic constraints on the diet of terrestrial carnivores. Nature.

[ref-7] Carbone C, Teacher A, Rowcliffe JM (2007). The costs of carnivory. PLOS Biology.

[ref-8] Carlson AA, Carlson R, Bercovitch FB (2004). African wild dog conservation project, Kafue National Park, Zambia. http://cres.sandiegozoo.org/projects/hc_wild_dogs_zambia.html.

[ref-9] Chansa W, Milanzi J (2010). Population status of the hippopotamus in Zambia. African Journal of Ecology.

[ref-10] Collados J, Garcia C, Rice CA (2018). Dental pathology of the Iberian lynx (*Lynx pardinus*) part 1: congenital, developmental, and traumatic abnormalities. Journal of Veterinary Dentistry.

[ref-11] Cozzi G, Börger L, Hutter P, Abegg D, Beran C, McNutt JW, Ozgul A (2015). Effects of trophy hunting leftovers on the ranging behaviour of large carnivores: a case study on spotted hyenas. PLOS ONE.

[ref-12] Creel S, Creel NM (2002). The African wild dog: behavior, ecology and conservation.

[ref-13] Creel S, Matandiko W, Schuette P, Rosenblatt E, Sanguinetti C, Banda K, Vinks M, Becker M (2018). Changes in African large carnivore diets over the past half-century reveal the loss of large prey. Journal of Applied Ecology.

[ref-14] Curtis AA, Orke M, Tetradis S, Van Valkenburgh B (2018). Diet-related differences in craniodental morphology between captive-reared and wild coyotes, *Canis latrans* (Carnivore: Canidae). Biological Journal of the Linnean Society.

[ref-15] De la Sancha NU, Boyle SA, Patterson BD (2017). Getting back to the basics: museum collections and satellite imagery are critical to analyzing species diversity. Bioscience.

[ref-16] DNPW (2016). Report on the 2015 aerial census of elephants and other large mammals in Zambia—part II: population estimates for other large mammals and birds.

[ref-17] Gregg E (2019). Zambia: leopard spotting in South Luangwa National Park—National Geographic 8. https://www.nationalgeographic.co.uk/travel/2018/08/zambia-leopard-spotting-south-luangwa-national-park.

[ref-18] Hayward MW (2006). Prey preferences of the spotted hyaena (*Crocuta crocuta*) and degree of dietary overlap with the lion (*Panthera leo*). Journal of Zoology.

[ref-19] Hayward MW, Kerley GIH (2005). Prey preferences of the lion (*Panthera leo*). Journal of Zoology.

[ref-20] IUCN (2006). Conservation strategy for the lion in Eastern and Southern Africa—IUCN SSC Cat Specialist Group. https://www.environment.gov.za/sites/default/files/docs/pantheraleo_conservationstrategy_regionalafrica.pdf.

[ref-21] IUCN (2016). African elephant status report 2016: an update from the African elephant database—Elephant Database: IUCN SSC, African Elephant Specialist Group—Gland, occasional paper of the IUCN Species Survival Commission No. 60. https://portals.iucn.org/library/node/46878.

[ref-22] Kruuk H (1972). The spotted hyena: a study of predation and social behavior.

[ref-23] Losey RJ, Jessup E, Nomokonova T, Sablin M (2014). Craniomandibular trauma and tooth loss in Northern dogs and wolves: implications for the archaeological study of dog husbandry and domestication. PLOS ONE.

[ref-24] Mann SA, Van Valkenburgh B, Hayward MW (2017). Tooth fracture within the African carnivore guild: the influence of intraguild competition and resource availability. Journal of Zoology.

[ref-25] Mech LD, Frenzel LD (1971). Ecological studies of the timber wolf in Northeastern Minnesota. Research Paper NC-52.

[ref-26] Miller JRB, Balme G, Lindsey PA, Loveridge AJ, Becker MS, Begg C, Brink H, Dolrenry S, Hunt JE, Janssoni I, Macdonald DW, Mandisodza-Chikerema RL, Cotterill AO, Packer C, Rosengren D, Stratford K, Trinkel M, White PA, Winterbach C, Winterbach HEK, Funston PJ (2016). Aging traits and sustainable trophy hunting of African lions. Biological Conservation.

[ref-27] Mitchell JCM, Shenton JB, Uys JCM (1965). Predation on large mammals in the Kafue National Park, Zambia. Zoologica Africana.

[ref-28] Mukherjee S, Heithaus MR (2013). Dangerous prey and daring predators: a review. Biological Reviews.

[ref-29] Purchase GK, Mateke C, Purchase D (2007). A review of the status and distribution of carnivores, and levels of human carnivore conflict, in the protected areas and surrounds of the Zambezi Basin: unpublished report—Bulawayo: The Zambezi Society. https://www.researchgate.net/publication/289344575.

[ref-30] Pyke GH, Pulliam HR, Charnov EL (1977). Optimal foraging: a selective review of theory and tests. The Quarterly Review of Biology.

[ref-31] Rausch RA (1967). Some aspects of the population ecology of wolves, Alaska. American Zoologist.

[ref-32] Ray RR (2011). Ecology and population status and the impact of trophy hunting of the leopard *Panthera pardus* (LINNAEUS, 1758) in the Luambe National Park and surrounding Game Management Areas in Zambia.

[ref-33] Rosenblatt E, Creel S, Becker MS, Merkle J, Mwape H, Schuette P, Simpamba T (2016). Effects of a protection gradient on carnivore density and survival: an example with leopards in the Luangwa Valley, Zambia. Ecology and Evolution.

[ref-34] Schaller GB, Schaller GB (1972). The Serengeti lion. Wildlife Behavior and Ecology Series.

[ref-35] Secretariat CITES (2016). Report on monitoring the illegal killing of elephants (MIKE)—(No. CoP17 Doc 57.5)—Geneva: CITES. https://cites.org/sites/default/files/eng/cop/17/WorkingDocs/E-CoP17-57-05.pdf.

[ref-36] Siamudaala VM, Nyirenda VR, Saiwana LM (2009). Effectiveness of law enforcement on wildlife crimes in the Kafue ecosystem in Zambia.

[ref-37] Smuts GL, Anderson JL, Austin JC (1978). Age determination of the African lion (*Panthera leo*). Journal of Zoology.

[ref-38] Smuts GL, Robinson GA, Whyte IJ (1980). Comparative growth of wild male and female lions (*Panthera leo*). Journal of Zoology.

[ref-39] Spong G, Hellborg L, Creel S (2000). Sex ratio of leopards taken in trophy hunting: genetic data from Tanzania. Conservation Genetics.

[ref-40] Stander PE (1997). Field age determination of leopards by tooth wear. African Journal of Ecology.

[ref-41] Tong H, Chen X, Zhang B, Rothschild B, White S, Balisi M, Wang X (2020). Hypercarnivorous teeth and healed injuries to *Canis chiliensis* from Early Pleistocene Nihewan beds, China, support social hunting for ancestral wolves. PeerJ.

[ref-42] Van Horn RC, McElhinny TL, Holekamp KE (2003). Age estimation and dispersal in the spotted hyena (*Crocuta crocuta*). Journal of Mammalogy.

[ref-43] Van Valkenburgh B (1988). Incidence of tooth breakage among large, predatory mammals. American Naturalist.

[ref-44] Van Valkenburgh B (1996). Feeding behavior in free-ranging large African carnivores. Journal of Mammalogy.

[ref-45] Van Valkenburgh B (2009). Costs of carnivory: tooth fracture in Pleistocene and recent carnivorans. Biological Journal of the Linnean Society.

[ref-46] Van Valkenburgh B, Hertel F (1993). Tough times at La Brea: tooth breakage in large carnivores of the late Pleistocene. Science.

[ref-47] Van Valkenburgh B, Peterson RO, Smith DW, Stahler DR, Vucetich JA (2019). Tooth fracture frequency in gray wolves reflects prey availability. eLife.

[ref-48] Van Valkenburgh B, Ruff CB (1987). Canine tooth strength and killing behaviour in large carnivores. Journal of Zoology.

[ref-49] Vucetich JA, Vucetich LM, Peterson RO (2012). The causes and consequences of partial prey consumption by wolves preying on moose. Behavioral Ecology and Sociobiology.

[ref-50] Wenker CJ, Stich H, Müller M, Lussi A (1999). A retrospective study of dental conditions of captive brown bears (*Ursus arctos spp*.) compared with free-ranging Alaskan grizzlies (*Ursus arctos horribilis*). Journal of Zoo and Wildlife Medicine.

[ref-51] White PA, Belant JL (2016). Individual variation in dental characteristics for estimating age of African lions. Wildlife Biology.

[ref-52] White PA, Diedrich CG (2012). Taphonomy story of a modern African elephant *Loxodonta africana* carcass on a lakeshore in Zambia (Africa). Quaternary International Mammoths and Their Relatives 2: Biotopes, Evolution and Human Impact.

[ref-53] White PA, Ikanda D, Ferrante L, Chardonnet P, Mesochina P, Cameriere R (2016). Age estimation of African lions *Panthera leo* by ratio of tooth areas. PLOS ONE.

[ref-54] White PA, Kim AJ (2018). A summary report and photographic catalogue of African wild dogs in the southern Kafue ecosystem, Zambia 2007–2012. Canid Biology and Conservation.

[ref-55] Wilkins L, Allen JM, Coltrain J, Flanagin S, Allen TD, Reed DL (2007). Methods of assessing health and diet of Florida panthers (*Puma concolor*) using museum specimens—part I: osteology as a method of assessing Florida panther health. Bulletin of the Florida Museum of Natural History.

[ref-56] Wobeser G (1992). Traumatic, degenerative, and developmental lesions in wolves and coyotes from Saskatchewan. Journal of Wildlife Diseases.

