# Supplementary material for: Naturally-occurring tooth wear, tooth fracture, and cranial injuries in large carnivores from Zambia
Source: PeerJ. 2021 Apr 20;9:e11313. doi: 10.7717/peerj.11313 (PMC8063872; doi:10.7717/peerj.11313)
Supplement: Supplemental Information 1 [file peerj-09-11313-s001.pdf]

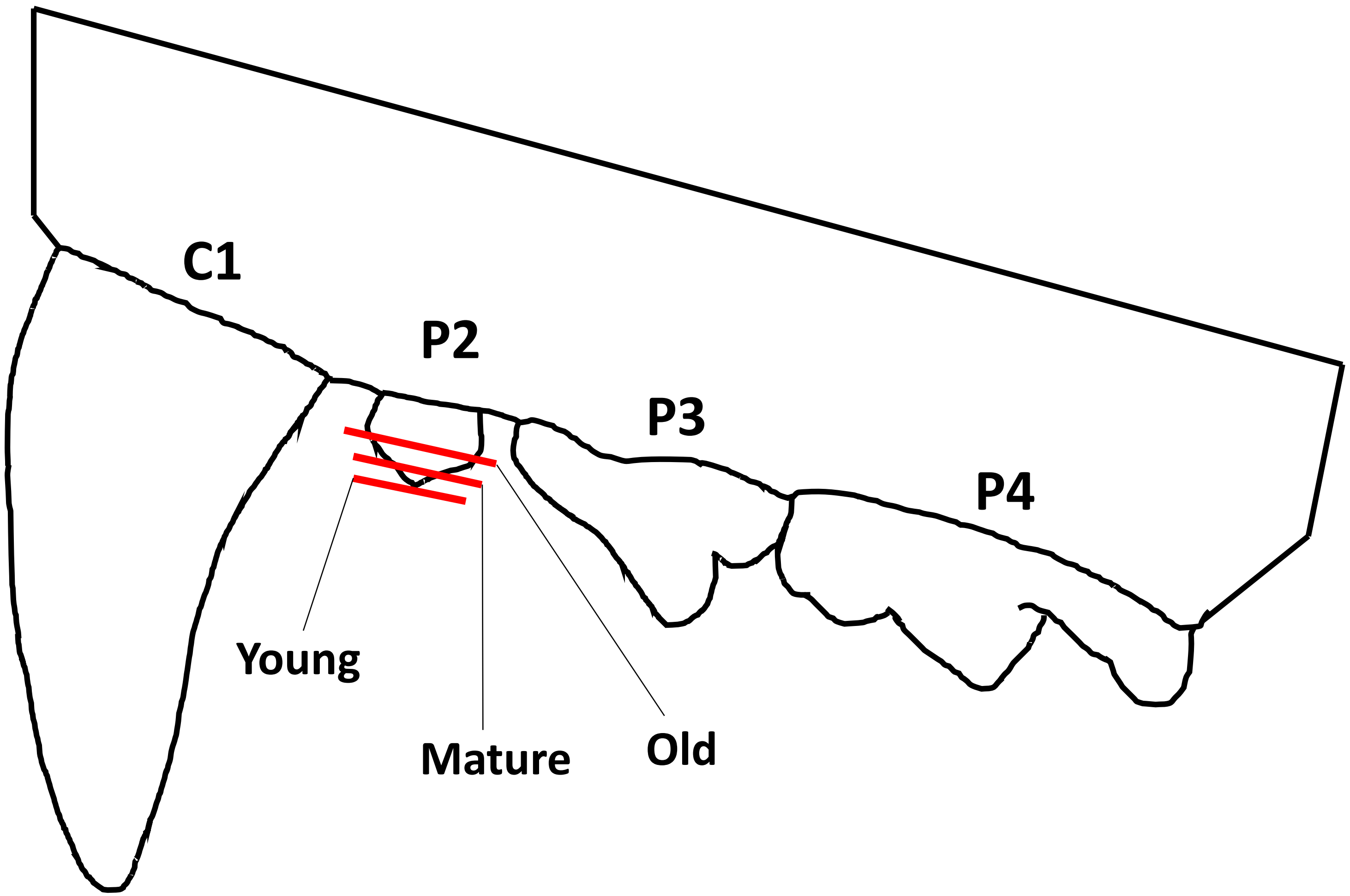

Age classes for Lion based on gradual rounding and flattening of the P2 as a result of normal wear (adapted from White and Belant 2016). Young: no wear/sharp; Mature: moderate wear/rounded; Old: heavy wear/flat,
