# Supplementary figures and images for: Naturally-occurring tooth wear, tooth fracture, and cranial injuries in large carnivores from Zambia
Source: PeerJ. 2021 Apr 20;9:e11313. doi: 10.7717/peerj.11313 (PMC8063872; doi:10.7717/peerj.11313)

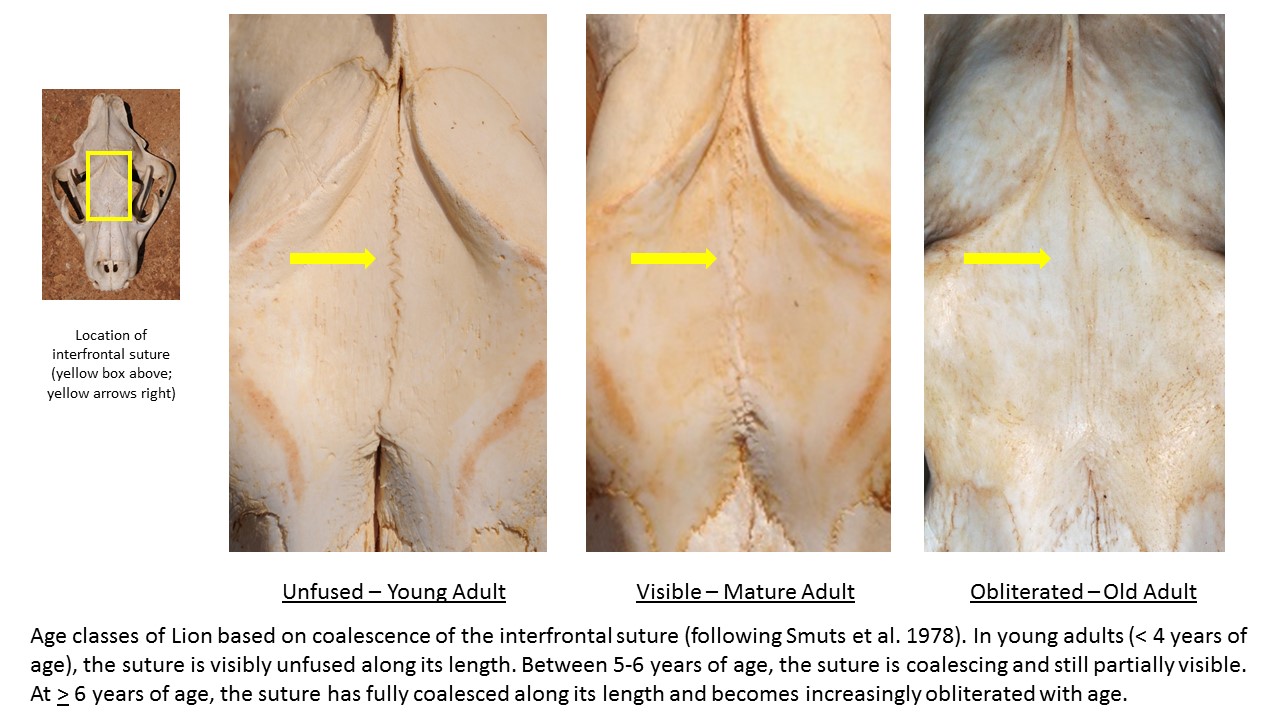

Supplement: Supplemental Information 2 [file peerj-09-11313-s002.jpg]

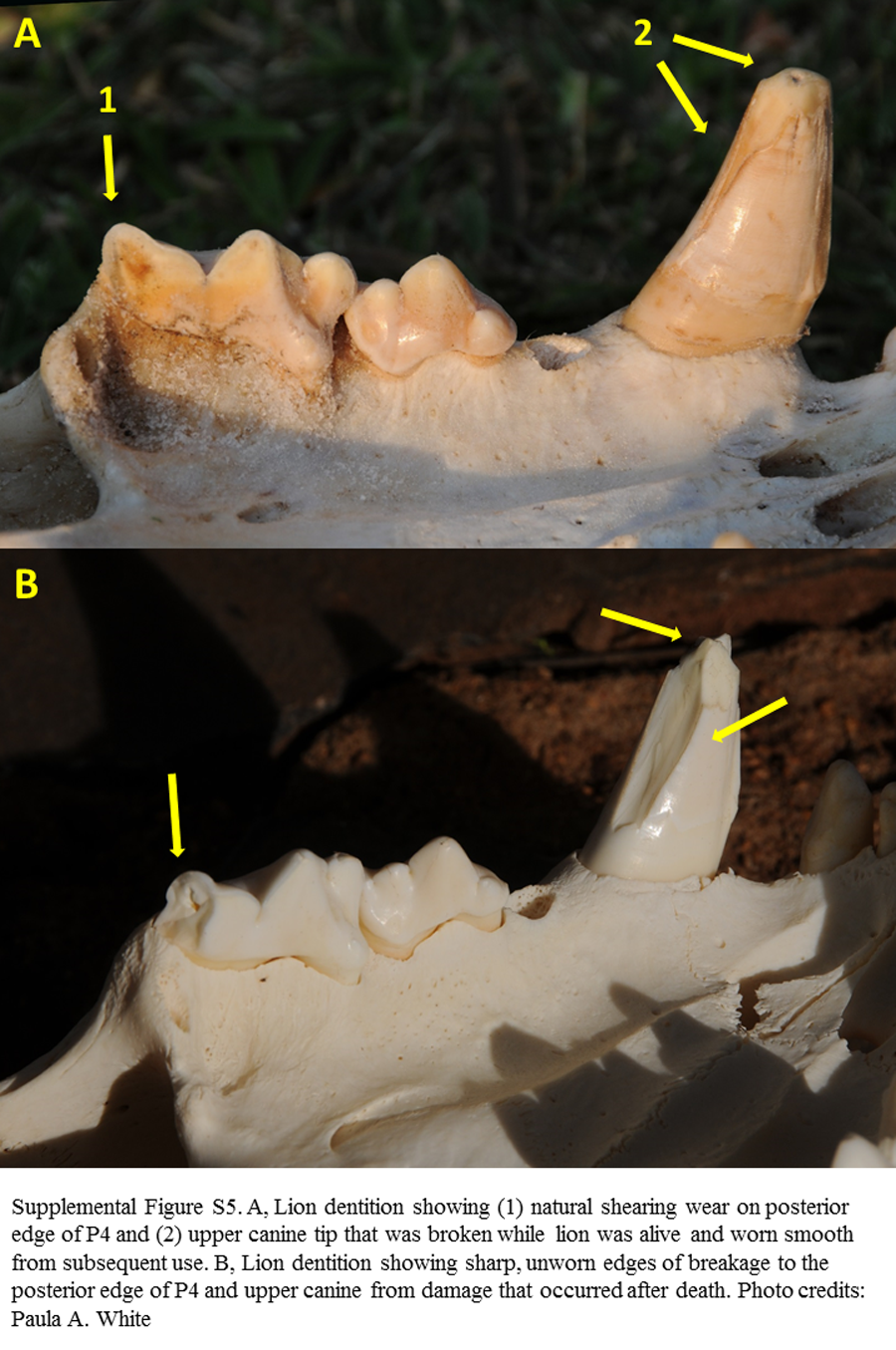

Supplement: Supplemental Information 5 — Supplemental Figure S5. (A) Lion dentition showing (1) natural shearing wear on posterior edge of P4 and (2) upper canine tip that was broken while lion was alive and worn smooth from subsequent use. (B) Lion dentition showing sharp, unworn edges of breakage to the posterior edge of P4 (arrow) and upper canine (arrow) from damage that occurred after death. Photo credits: Paula A. White [file peerj-09-11313-s005.png]

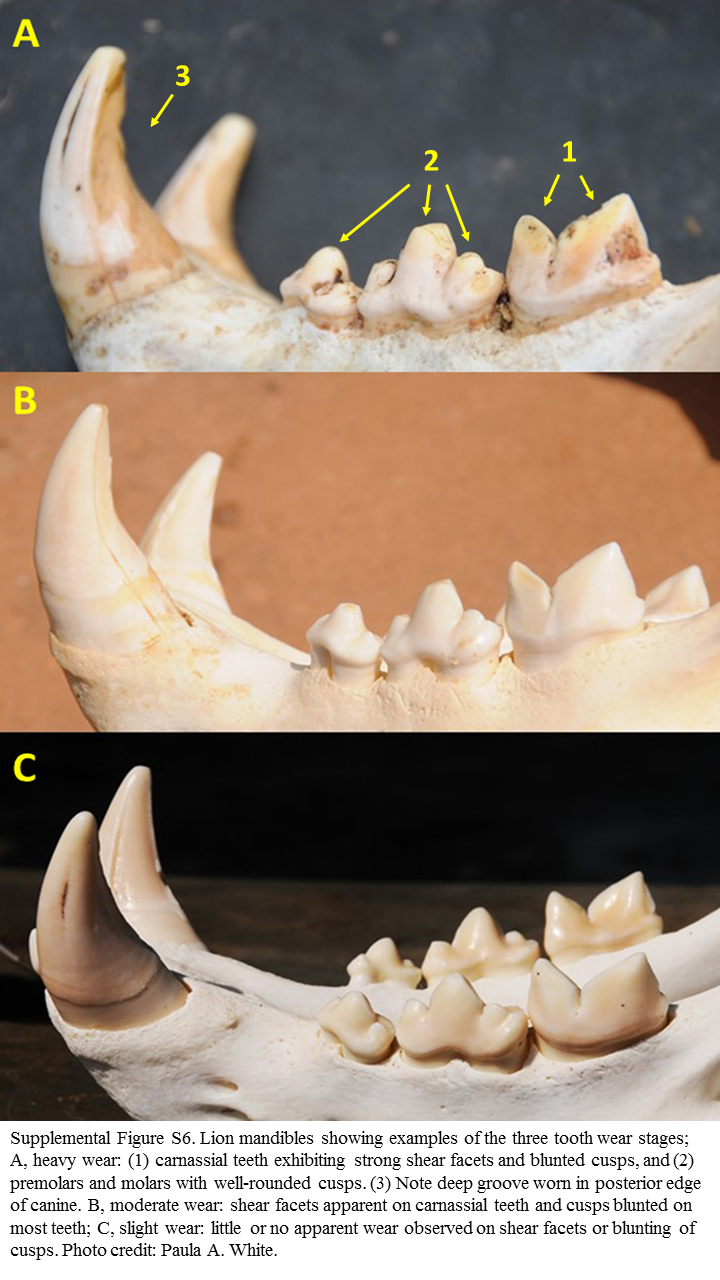

Supplement: Supplemental Information 6 — Supplemental Figure S6. Lion mandibles showing examples of the three tooth wear stages. (A) Heavy wear. (B) Moderate wear. (C) Slight wear. Photo credit: Paula A. White. [file peerj-09-11313-s006.png]
