# Supplementary material for: Naturally-occurring tooth wear, tooth fracture, and cranial injuries in large carnivores from Zambia
Source: PeerJ. 2021 Apr 20;9:e11313. doi: 10.7717/peerj.11313 (PMC8063872; doi:10.7717/peerj.11313)
Supplement: Supplemental Information 3 [file peerj-09-11313-s003.pdf]

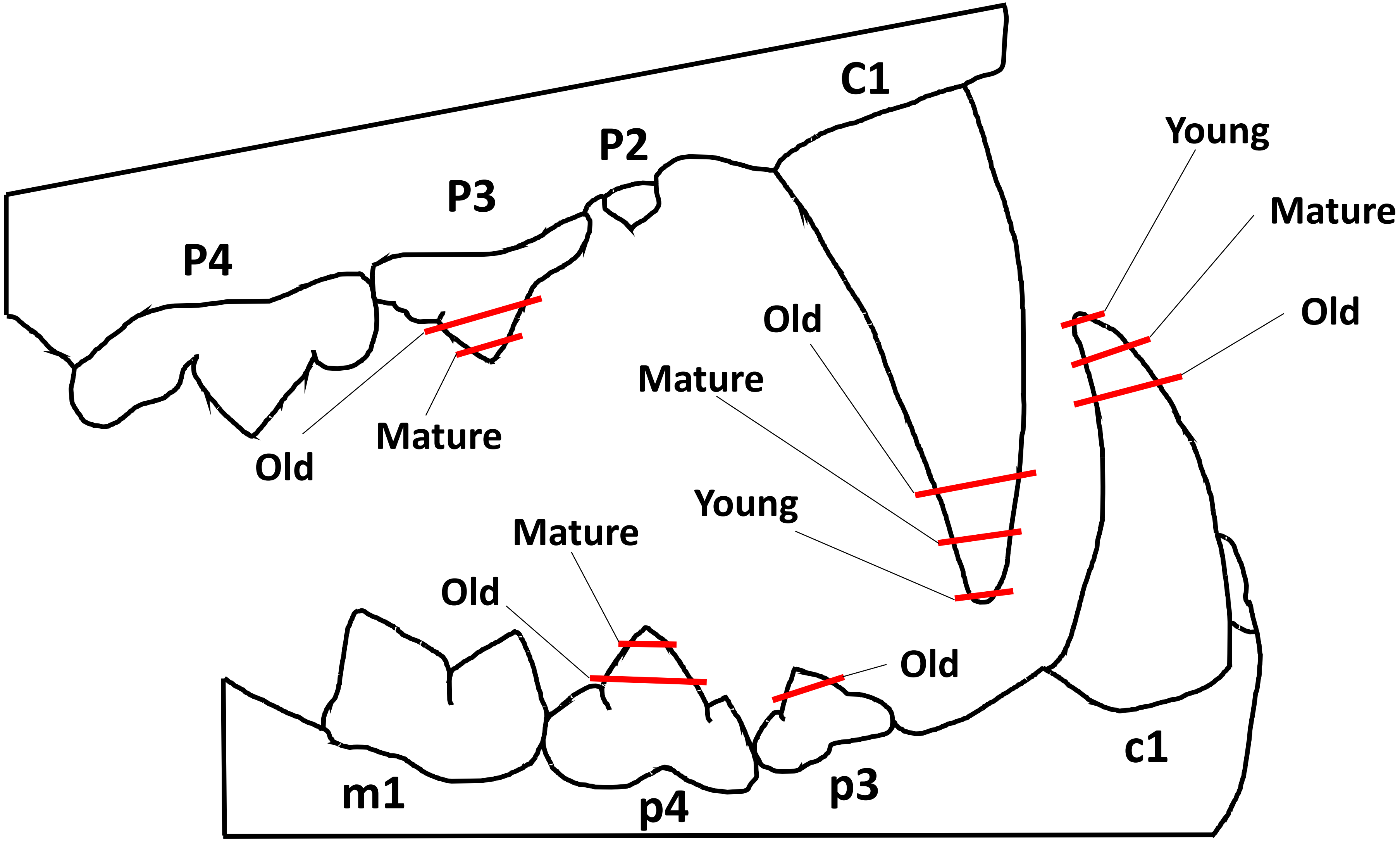

Age classes for Leopard based on gradual rounding and flattening of the C1, c1, P3, p3,4 as a result of normal wear (adapted from Stander 1997). Young adult ≤ 4 years; Mature adult 5-6 years; Old adult 7-10 years.
