# Supplementary material for: Naturally-occurring tooth wear, tooth fracture, and cranial injuries in large carnivores from Zambia
Source: PeerJ. 2021 Apr 20;9:e11313. doi: 10.7717/peerj.11313 (PMC8063872; doi:10.7717/peerj.11313)
Supplement: Supplemental Information 4 [file peerj-09-11313-s004.pdf]

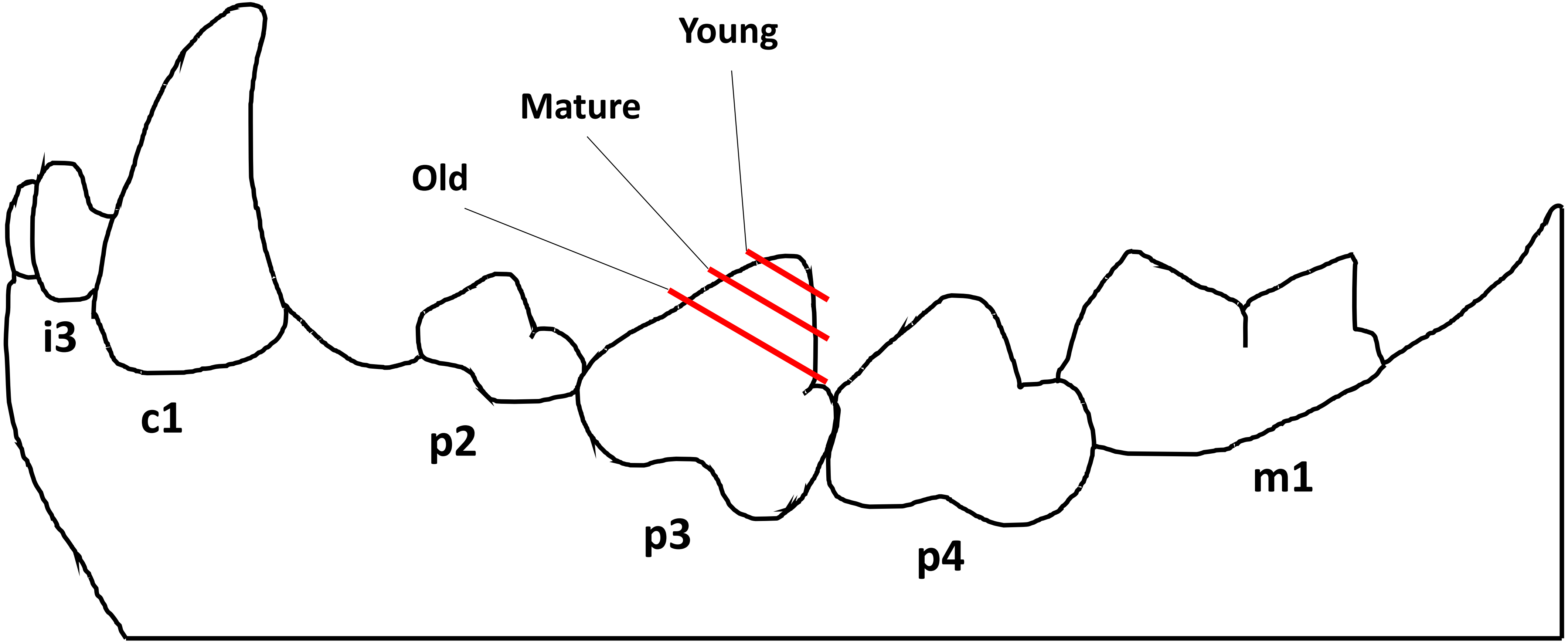

Age classes for Spotted Hyena based on gradual flattening of the occlusal surface of the p3 as a result of normal wear (adapted from Kruuk 1972). Young 1-3 years; Mature >3-6 years; Old >6-16 years.
