# Supplementary material for: Naturally-occurring tooth wear, tooth fracture, and cranial injuries in large carnivores from Zambia
Source: PeerJ. 2021 Apr 20;9:e11313. doi: 10.7717/peerj.11313 (PMC8063872; doi:10.7717/peerj.11313)
Supplement: Supplemental Information 7 — Total number of teeth present for each tooth position and percent broken in parentheses for individuals from Luangwa Valley (LV) and the Greater Kafue Ecosystem (GKE). [file peerj-09-11313-s007.docx]

| **SAMPLE** | **N**  **Incisors**  **(% brkn)** | **N**  **CANINES**  **(% brkn)** | **N**  **PREMOLARS**  **(% brkn)** | **N**  **CARNASSIALS**  **(% brkn)** | **N**  **PC molars**  **(% brkn)** |
| --- | --- | --- | --- | --- | --- |
| *Panthera leo* |  |  |  |  |  |
| LV | 675 (0.083) | 231  (0.048) | 427  (0.021) | 230  (0.026) | 95  (0.010) |
|  |  |  |  |  |  |
| GKE | 658  (0.052) | 228  (0.022) | 421  (0.005) | 227  (0.004) | 94  (0) |
|  |  |  |  |  |  |
| *Panthera pardus* |  |  |  |  |  |
| LV | 211  (0.024) | 72  (0.069) | 142  (0) | 72  (0) | 20  (0) |
|  |  |  |  |  |  |
| GKE | 318  (0.028) | 108  (0.056) | 211  (0.005) | 108  (0.019) | 52  (0) |
|  |  |  |  |  |  |
|  |  |  |  |  |  |
| *Crocuta crocuta* | 147  (0.075) | 52  (0.231) | 152  (0.112) | 51  (0.137) |  |
